# Organ of Corti and Stria Vascularis: Is there an Interdependence for Survival?

**DOI:** 10.1371/journal.pone.0168953

**Published:** 2016-12-28

**Authors:** Huizhan Liu, Yi Li, Lei Chen, Qian Zhang, Ning Pan, David H. Nichols, Weiping J. Zhang, Bernd Fritzsch, David Z. Z. He

**Affiliations:** 1 Department of Biomedical Sciences, Creighton University School of Medicine, Omaha, Nebraska, United States of America; 2 Department of Otorhinolaryngology, Beijing Tongren Hospital, Beijing Capital Medical University, Beijing, China; 3 Chongqing Academy of Animal Science, Chongqing, China; 4 Department of Biology, University of Iowa, Iowa City, Iowa, United States of America; 5 Department of Pathophysiology, Second Military Medical University, Shanghai, China; University of South Florida, UNITED STATES

## Abstract

Cochlear hair cells and the stria vascularis are critical for normal hearing. Hair cells transduce mechanical stimuli into electrical signals, whereas the stria is responsible for generating the endocochlear potential (EP), which is the driving force for hair cell mechanotransduction. We questioned whether hair cells and the stria interdepend for survival by using two mouse models. *Atoh1* conditional knockout mice, which lose all hair cells within four weeks after birth, were used to determine whether the absence of hair cells would affect function and survival of stria. We showed that stria morphology and EP remained normal for long time despite a complete loss of all hair cells. We then used a mouse model that has an abnormal stria morphology and function due to mutation of the *Mitf* gene to determine whether hair cells are able to survive and transduce sound signals without a normal electrochemical environment in the endolymph. A strial defect, reflected by missing intermediate cells in the stria and by reduction of EP, led to systematic outer hair cell death from the base to the apex after postnatal day 18. However, an 18-mV EP was sufficient for outer hair cell survival. Surprisingly, inner hair cell survival was less vulnerable to reduction of the EP. Our studies show that normal function of the stria is essential for adult outer hair cell survival, while the survival and normal function of the stria vascularis do not depend on functional hair cells.

## Introduction

Hearing depends on normal function of the organ of Corti and stria vascularis in the cochlea. The organ of Corti, situated on the basilar membrane in the scala media of the cochlea, contains two types of hair cells, inner hair cells (IHCs) and outer hair cells (OHCs). Both types of hair cells are able to transduce mechanical signals through mechanosensitive transduction channels in the stereocilia bundle on their apical surfaces. The lateral wall of the scala media is composed of the outer spiral sulcus, stria vascularis, and spiral ligament. The stria vascularis, composed of marginal, intermediate, and basal cells, is responsible for maintaining the ion composition of the endolymph and producing an endocochlear potential (EP) in the scala media [1,2]. Recent physiological models for the mechanism of EP generation suggest that K^+^ channels/pumps and intermediate cells play an important role in the generation of EP and K^+^ transport [3–5]. It is generally assumed that there is a K^+^ recycling pathway that involves the stria vascularis, hair cells, supporting cells, and fibrocytes of the spiral ligament [4,6]. In this model, K^+^ ions in the endolymph are driven into hair cells by the sum of the EP and the resting membrane potential of the hair cells via the transduction channel and out into the perilymph via basolateral K^+^ channels. They are then taken up by supporting cells. After passing through gap-junction channels between supporting cells, K^+^ ions enter the perilymph, where they are taken up by fibrocytes of the spiral ligament, then basal cells [7]. They then diffuse into strial intermediate cells. The final step in K^+^ recycling is the actual generation of the EP, when K^+^ ions are released from the intermediate cells into the intrastrial space via the Kcnj10 K^+^ channel [8,9]. This K^+^ ion recycling pathway is one of the mechanisms that maintains the EP and ion homeostasis in the endolymph [3,4,10]. Consistent with this model is that over 60% of congenital hearing loss involves mutations of gap junction proteins thought to disrupt the K^+^ circulation [3,4].

Studies in the 1980s and 1990s established the importance of the EP for auditory sensitivity and frequency selectivity. Those studies demonstrated that, when K^+^ concentration in the endolymph or the EP magnitude is reduced by locally or intravenously applied diuretic compounds such as furosemide [11], basilar membrane vibration is reduced at the characteristic frequency [12], and the threshold of tuning curves of auditory nerve fibers at the characteristic frequency is elevated [13,14]. Interestingly, while the electrochemical environment in the endolymph and the EP maintained by the stria has long been known to be important for hair cell mechanotransduction and cochlear amplification, it is unclear whether reduction or loss of the EP would affect hair cell survival.

The presence of K^+^ recycling via the organ of Corti implies that the hair cells and supporting cells are integrated into the function of the stria and maintenance of the EP. However, it has not been determined whether hair cell abnormality or loss would lead to degeneration or malfunction of the stria as their absence should eliminate the K^+^ reentry into the epithelium for epithelial recirculation to the stria. Understanding causally these relationships between hair cells and the stria through genetic defects in either, but not both, would be important for understanding the basic ionic balance regulatory mechanism in the cochlea and thus the pathology of hearing loss associated with abnormal hair cells (such as Usher syndrome) or stria vascularis (such as Waardenburg syndrome and Tietz syndrome). Therefore, the goal of our study is to determine whether disruption or abnormality of either hair cells or the stria alone would lead to malfunction or degeneration of the other in a mutually interdependent way.

Two mouse models, *Atoh1* conditional knockout (*Atoh1*-CKO) mice [15] and *microphthalmia-associated transcription factor* gene (*Mitf*) mutant mice [16], were used for our study. Atoh1, a transcription factor belonging to the family of basic helix-loop-helix (bHLH)-containing proteins, plays a critical role for hair cell differentiation and survival during development and adulthood [15,17,18]. The *Atoh1*-CKO mouse self-terminates expression of Atoh1 in hair cells around birth using an Atoh1 enhancer to drive cre to conditionally delete the floxed *Atoh1*. *Atoh1*-CKO mice display a complete loss of hair cells within three weeks after birth due to the self-termination of Atoh1 expression [15]. We used this mouse model to determine whether absence of hair cells and the organ of Corti would affect development, function, and survival of the stria. The second mouse model has a mutation of the *Mitf* gene expressed in melanocytes in the stria vascularis. Mitf is a basic helix-loop-helix leucine zipper transcription factor involved in melanocyte and osteoclast development [19,20]. Loss of melanocytes due to *Mitf* mutation causes abnormality of the stria morphology and function, leading to hearing loss in both humans and mice [16,21,22]. *Mitf* mutation in humans is one of the causes of Waardenburg syndrome [23], a group of genetic conditions that can cause hearing loss and change in coloring of the hair, skin, and eyes [24]. We used this mouse model to determine whether a change in the electrochemical environment (i.e., reduction of EP) in the endolymph maintained by the stria would affect function and survival of hair cells. Understanding such a relationship between the organ of Corti and stria is important for understanding the basic mechanism of hearing and the pathology of congenital hearing loss associated with an abnormal stria vascularis (such as in Waardenburg syndrome and Tietz syndrome) or abnormal hair cells (as in Usher Syndrome). Understanding such relationship also provides guidance for exploring therapeutic strategy by restoring the EP for preventing hair cell loss in patients with Warrdenburg syndrome or Tietz syndrome.

## Materials and Methods

### Mouse models

Wild-type (WT), heterozygous, and homozygous mice were used for the study. The *Atoh1-cre;Atoh1*^*f/f*^ conditional knockout, referred to as *Atoh1*-CKO mice, were generated by breeding mice carrying the *Atoh1-cre* transgene with mice carrying the floxed *Atoh1* gene [15]. *Atoh1-cre* transgene mice, defined as WT mice with no floxed *Atoh1* allele, were used as controls to verify that cre expression itself is not causing any alterations in hair cells. *Mitf*-mutant mice (C57BL/6-*Mitf*^*Mi‐wh/+*^) were purchased from Jackson Laboratory (Bar Harbor, ME, USA). C57BL/6-*Mitf*^Mi-wh/Mi-wh^ homezygotes, C57BL/6-*Mitf*^Mi-wh/+^ heterozygotes, and their C57BL/6-^+/+^ littermates were used for the experiments [16]. Care and use of the animals in this study was approved by the Institutional Animal Care and Use Committees of Creighton University and the University of Iowa.

### Hearing threshold measurement using auditory brainstem responses (ABR)

Five animals for each mouse strain were used for ABR recordings. The mouse was anesthetized with a mixture of ketamine/xylazine (ketamine 100 mg/kg; xylazine 15 mg/kg; ip), and supplemented as needed. ABRs were recorded in response to tone bursts of 2, 4, 8, 16, 22, and 32 kHz using standard procedures previously described [25,26]. Tone pipe with 1 ms rise cosine on/off ramps were generated digitally using a clock rate of 125 kHz and 16-bit D/A converters. Stimulus levels were calibrated using a 1/8 inch B & K microphone (Model 4138) and were presented as sound pressure levels measured in decibels (dB SPL: referenced to 20 mPa). ABR signals were collected with subcutaneous platinum needle electrodes placed at the vertex, mastoid prominence, and shoulder. Response signals were amplified (100,000x), filtered, and acquired by TDT Workstations (Tucker-Davis Technologies). Each averaged response was based on 200 stimulus repetitions. During the procedure, the body temperature was maintained at 37°C with a heating pad. All records were obtained in a sound-attenuating chamber.

### Recording of cochlear microphonic (CM) and EP

After mice were anesthetized, an incision was made in the inferior portion of the right postauricular sulcus. The bulla was perforated allowing for exposure of the stapedial artery, basal turn, and apical turn of the cochlea. A silver electrode was placed on the ridge near the round window for recording CM [25]. The ground electrode was implanted in the dorsal neck muscles. Tone bursts with different frequencies (2, 4, or 8 kHz) and amplitude were delivered through a calibrated TDT MF1 multi-field magnetic speaker. The biological signals (filtered at different frequencies) were amplified under current-clamp mode using an Axopatch 200B amplifier (Molecular Devices, Sunnyvale, CA, USA) and acquired by software pClamp 9.2 (Molecular Devices) running on an IBM-compatible computer with a 16-bit A/D converter (Digidata 1322B, Molecular Devices). The sampling frequency was 25 to 50 kHz.

For recording the EP, a basal turn location was chosen. This location was approximately 2 mm from the basal end, corresponding to the region with best frequency of 20–30 kHz, based on the cochlear frequency map for the mouse [27]. A hole was made using a fine drill on the cochlear wall. A glass capillary pipette electrode (10–20 MΩ) filled with 150 mM KCl was mounted on a hydraulic micromanipulator and advanced until a stable positive potential was observed. The ground electrode was implanted in the dorsal neck muscles. The voltage change during penetration was continuously recorded under the gap-free model using the Clampex in the pClamp software package. The signals were filtered and amplified under current-clamp mode using an Axopatch 200B amplifier and acquired by software pClamp 9.2. The sampling frequency was 10 to 25 kHz. Data were analyzed using Clampfit and Igor Pro (WaveMetrics, Portland, OR, USA).

### Hair cell counting and morphology of the stria vascularis

The cochleae were perfused with 4% paraformaldehyde (PFA) after transcardiac perfusion and maintained in the fixative at 4°C overnight. The basilar membrance, including the organ of Corti, was dissected and cut into apical and basal segments. After several washes with PBS, the tissue was blocked for 1 hour with 0.25% normal goat serum in PBS containing 0.01% Triton-X-100. Primary antibody for Myo7a (Invitrogen) was diluted 1:200 and incubated for 24 hours at 4°C. After washes with PBS, secondary antibody (1:500) (Alexa fluor molecular probe 488; Invitrogen) was added and incubated overnight at 4°C. Tissues were washed with PBS and mounted on glass microscopy slides with antifade solution (5 ml PBS, 5 ml glycerol, 0.1 g n-propylgallate). Images were captured using a LSM 510 META confocal scanning system with three lasers mounted on a Zeiss AxioPlan 2IE MOT motorized upright microscope (Carl Zeiss International). Hair cell counts from two areas (each 400 μm in length) at the basal and apical turns were obtained from confocal images off-line as described before [26].

For examination of stria morphology [28], the fixed cochleae were embedded in celloidin. Serial cross-sections (~10 μm) were prepared with a microtome (CM3050, Leica, Germany) and collected on microslides. The sections were stained with hematoxylin and eosin (H & E) and then washed. The slides were mounted with glycerol and examined under a microscope. Images at 10x and 25x magnification were taken. The width of the SV was determined using ImageJ (version 1.47) from captured images. For all statistical analysis, a two-tailed student’s t-test was used and p < 0.05 was considered significant.

### Measurement of nonlinear capacitance (NLC)

OHCs were isolated using enzymatic digestion and gently stirred using a pipette with a large bore and bathed in an extracellular solution containing (in mM) 120 NaCl, 20 TEA-Cl, 2 CoCl_2_, 2 MgCl_2_, 10 HEPES, and 5 glucose, at pH 7.3. The internal solution contained 140 CsCl, 2 MgCl_2_, 10 EGTA, and 10 HEPES at pH 7.3 to block voltage-dependent ion channels in the basolateral membrane, thereby isolating the capacitive current. After whole-cell voltage-clamp recording was established at room temperature (20±2°C), a two-sine voltage stimulus protocol (10 mV peak at both 390.6 and 781.2 Hz) with subsequent fast Fourier transform-based admittance analysis was used to measure membrane capacitance [29] from a holding potential of 0 mV. The capacitive currents were sampled at 100 kHz and low-pass filtered at 5 kHz. Series resistance was compensated off-line. Data were acquired using jClamp (Scisoft, New Haven, CT) and analyzed with Igor (WaveMetrics, Portland, OR).

The NLC can be described as the first derivative of a two-state Boltzmann function relating nonlinear charge movement to voltage [30,31]. The capacitance function is described as:

$$C_{m}=\frac{Q_{\text{max}}\alpha }{\text{exp}[\alpha (V_{m}-V_{1/2})]{\left(1+\text{exp}[-\alpha (V_{m}-V_{1/2})]\right)}^{2}}+C_{lin}^{}$$

where *Q*_*max*_ is maximum charge transfer, *V*_*1/2*_ is the voltage at which the maximum charge is equally distributed across the membrane, or equivalently, the peak of the voltage-dependent capacitance, *C*_*lin*_ is linear capacitance, and *α* = *ze/kT* is the slope of the voltage dependence of charge transfer where *k* is Boltzmann’s constant, *T* is absolute temperature, *z* is valence of charge movement, and *e* is electron charge. When resting membrane potential was measured under the voltage-clamp condition, normal intracellular and extracellular solutions were used [32,33].

## Results

### Hearing threshold and hair cell loss in *Atoh1*-CKO mice

**Fig 1:**
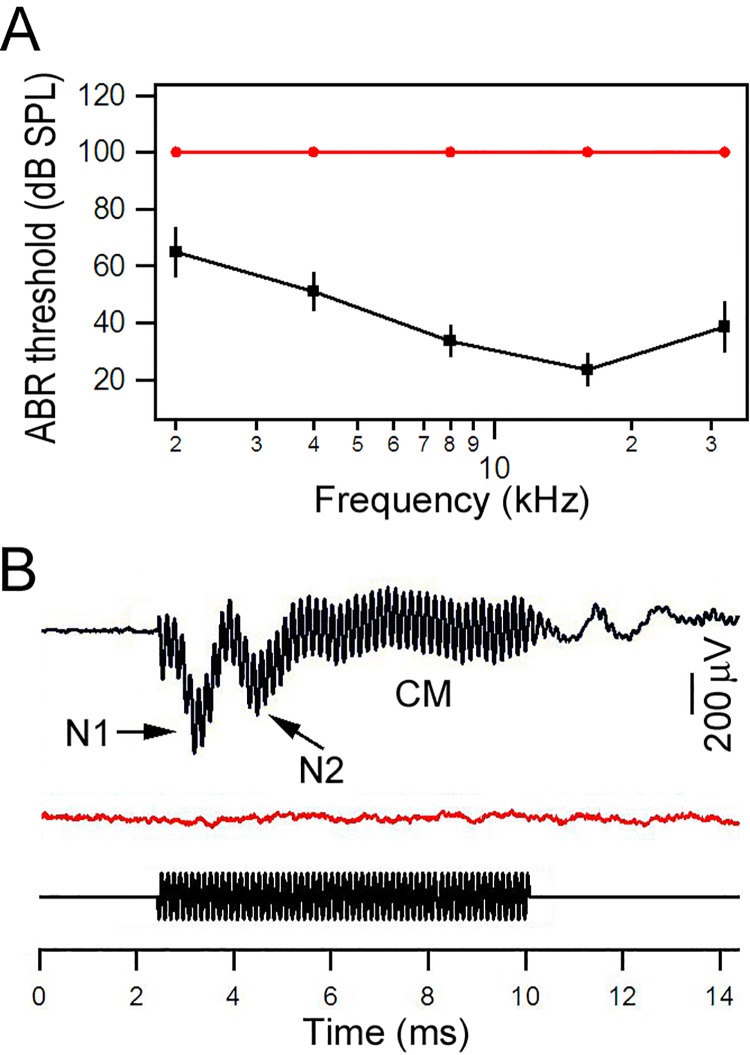
Auditory functions measured from WT and *Atoh1*-CKO mice. **A**: ABR thresholds obtained from 2-month-old WT (in black) and *Atoh1*-CKO (in red) mice. **B**: Examples of CM and CAP obtained from WT and *Atoh1*-CKO mice using an 8 kHz tone burst (bottom trace). N1 and N2 represent the two components from the CAP. No CM or CAP responses were seen from the *Atoh1*-CKO mouse.

We measured ABR thresholds and CMs from *Atoh1*-CKO mice and their WT littermates at two months of age. This is because a complete loss of hair cells was expected at this age. This is based on the study by Pan et al. [15], who showed that a progressive loss of hair cells from the base to the apex begins in late embryos, and a complete loss of basal-turn hair cells and a near complete loss of apical-turn hair cells are observed by P38. We measured tone pipe-evoked ABRs at 2, 4, 8, 16, 22, and 32 kHz to determine hearing threshold, Fig 1A shows the mean ABR thresholds from five *Atoh1*-CKO and five WT mice. No positive ABR response was detected at 100 dB SPL (the highest intensity) in *Atoh1*-CKO mice. We also measured CM and compound action potential (CAP) using the round window electrode technique. CM, an electrical potential generated in the cochlear hair cells in response to acoustic stimulation, primarily reflects mechanotransduction in the stereocilia of OHCs [34]. An example of CM and CAP evoked by a 8-kHz tone burst (80 dB) from a WT mouse is exhibited in Fig 1B. In *Atoh1*-CKO mice, neither CM nor CAP was observed with 100 dB SPL tones at 2, 4, or 8 kHz. An example of lack of CM and CAP response is shown in the middle panel of Fig 1B.

**Fig 2:**
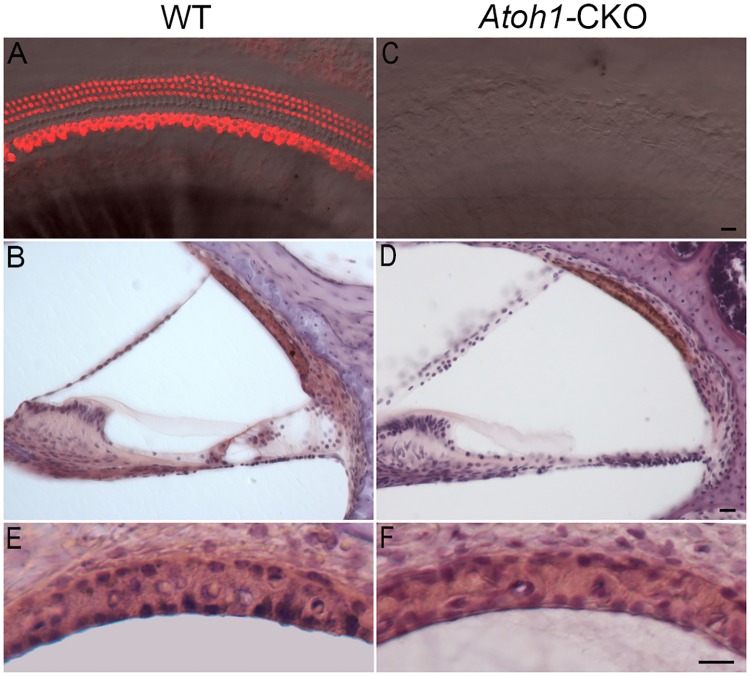
Representative images of the organ of Corti and lateral wall of the scala media of 2-month-old WT and *Atoh1*-CKO mice. **A** and **C**: Images of hair cells in the lower apical turn of WT and *Atoh1*-CKO mice. The images are from superimposed confocal and transmission light microscopic pictures taken from the same location in same tissues. Hair cells were labeled with anti-Myo7a antibody. Bar: 10 μm for A and C. **B** and **D**: Cross-section images of the scala media of the lower apical turn from 2-month-old WT and *Atoh1*-CKO mice. Bar: 10 μm for B and D. **E** and **F**: Magnified images of the stria vascularis from WT and *Atoh1*-CKO mice. Bar: 10 μm for E and F.

We used confocal microscopy to examine the status of hair cells in the apical turn of 2-month-old *Atoh1*-CKO and WT mice by labeling hair cells with anti-Myo7a antibody in whole mount preparations. We chose to examine the apical turn because hair cells there were the last to be lost in the *Atoh1*-CKO mice [15]. Therefore, absence of hair cells in the apical turn would signify a complete loss of hair cells in the entire cochlea. As shown in Fig 2A, three rows of OHCs and one row of IHCs are observed in the WT mouse cochlea. Similarly, three rows of OHCs and one row of IHCs together with supporting cells were also observed in the cochlear section preparation (Fig 2B). In *Atoh1*-CKO mice, no Myo7a-positive cells were observed in the entire length of the cochlea and the organ of Corti was replaced by a flat epithelium consisting of BMP4 positive Claudius cells [15,35]. An example of the lack of Myo7a-positive cells from the apical turn is shown in Fig 2C. Cochlear sectioning showed that the organ of Corti completely disappeared and was replaced by flat epithelial cells (Fig 2D).

### Stria morphology and function in *Atoh1*-CKO mice

We examined stria morphology and function in 2-month-old *Atoh1*-CKO mice when no sensory epithelium was present on the basilar membrane. Absence of the sensory hair cells and supporting cells in the organ of Corti allowed us to determine whether strial function and survival are dependent on normal function of the organ of Corti. We first examined gross morphology of the stria vascularis using thin sections of the cochleae. Fig 2C and 2F shows two images of the stria obtained from the cochlea of a WT and an *Atoh1*-CKO mouse. In both images, three layers of cells in the stria can be seen. We measured the width of the stria in the apical and basal turns from three mice for each genotype using ImageJ software. The width of the stria in the apex and base was 23.1 ± 1.5 and 23.8 ± 1.3 μm, respectively, for the WT mice, whereas that of the *Atoh1*-CKO mice was 23.5 ± 1.2 and 23.5 ± 1.3 μm, respectively. We compared the width of the stria from the same location of the cochleae between WT and *Atoh1*-CKO mice. No statistical significant difference (*p* = 0.37 and 0.36 for the apex and base, respectively) was found.

**Fig 3:**
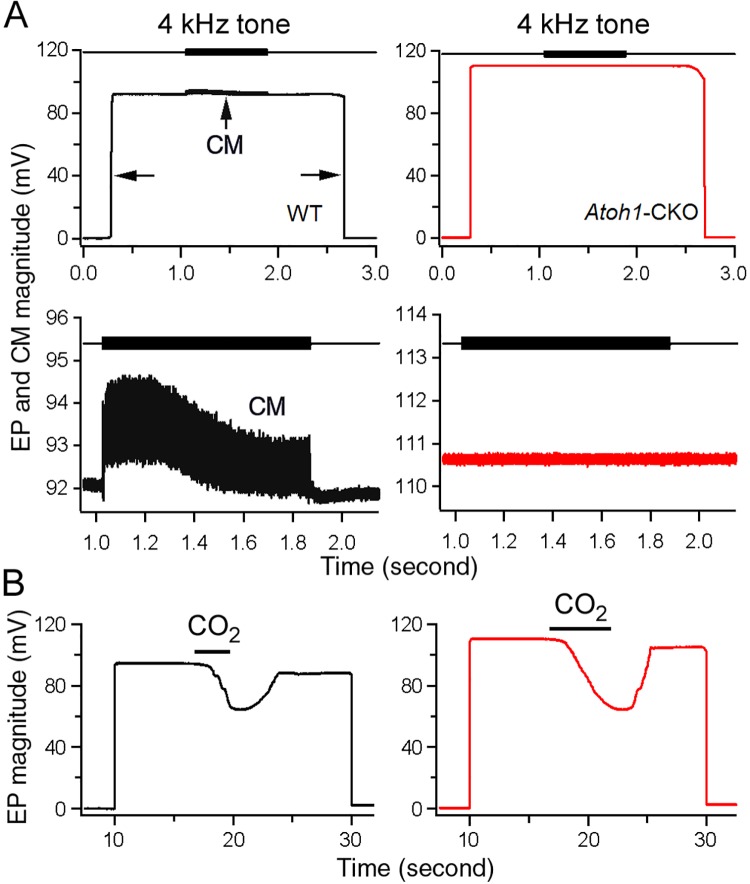
EP and CM recorded from WT and *Atoh1*-CKO mice. **A**: Representative waveforms of EP and CM recorded from 2-month-old WT (in black) and *Atoh1*-KO (in red) mice. Black arrows indicate the time points when a sharp electrode penetrated into and withdrawn from the scala media. A 4 kHz tone burst (80 dB SPL) was presented during EP measurement. CM with peak-to-peak magnitude of ~2 mV was observed from the WT mouse. However, no CM was seen from the *Atoh1*-CKO mouse. The CM response with expanded scale was plotted in the middle panels. **B**: Change of EP magnitude during CO_2_ inhalation in 13-month-old WT and *Atoh1*-KO mice. The bars indicate duration of CO_2_ inhalation.

We measured the EP from *Atoh1*-CKO mice to determine whether loss of the organ of Corti would affect stria function and survival. The microelectrode technique was used to measure the EP in the basal turn through the lateral wall approach. In WT littermates, a 94 mV potential was observed when the electrode penetrated into the scala media (Fig 3A). When a 4 kHz tone burst was presented during EP recording, a cycle-by-cycle response (marked as CM in Fig 3A) was evoked. This ~2 mV potential with a dc component (summating potential) is from the extracellular receptor potential of hair cells [34]. We measured the EP from the basal turn of six WT mice at P60. The mean magnitude was 94 ± 3.9 mV, consistent with previous EP values for mice [36]. Interestingly, the EP of *Atoh1*-CKO mice was ~110 mV (right panel of Fig 3A), with a mean magnitude of 113 ± 4.8 mV (n = 6). This mean value was significantly greater (*p* = 1.2e-5) than that of the WT control. During tone burst presentation, no cycle-by-cycle response was observed (right panels in Fig 3B), consistent with the morphological observation that no hair cells or organ of Corti were present in *Atoh1*-CKO mice. Thus, the EP remained in *Atoh1*-CKO mice despite a complete loss of hair cells. We examined the long-term consequence of absence of the organ of Corti to the EP in *Atoh1*-CKO mice at the age of 13 months. The EP magnitude (114 ± 4.5 mV, n = 6) remained unchanged (*p* = 0.38) when compared with that at P60 (113 ± 4.8 mV, n = 6).

The EP is highly dependent on metabolism and ion transport [37–39] and recirculation of K^+^ ions form the organ of Corti to the stria [4]. The reduction in EP magnitude and its ability to rapidly recover after anoxia stops are often used as a strong indication of a functional stria. We examined stria function by monitoring the change of EP magnitude during and after intake of CO_2_ in WT and *Atoh1*-*CKO* mice at 13 months of age. As shown in Fig 3B, the EP magnitude is reduced during CO_2_ inhalation and quickly recovers when CO_2_ is stopped in WT and *Atoh1*-CKO mice. The vulnerability to anoxia and the ability to recover seen in *Atoh1*-CKO mice indicate that their stria was functional after hair cells and an organ of Corti were long gone.

### Hearing threshold and hair cell loss in *Mitf*-mutant mice

**Fig 4:**
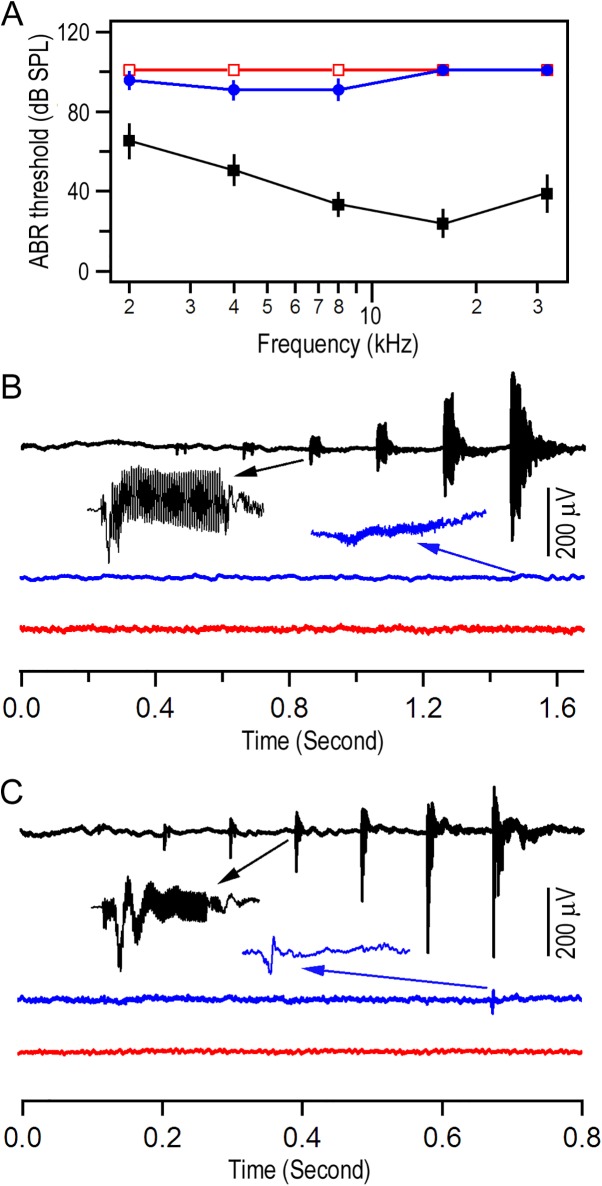
Auditory function of *Mitf*-mutant mice. **A**: ABR thresholds of WT, heterozygous, and homozygous mice. **B**: CM evoked by 2 kHz tone bursts with increasing intensity from 30 to 90 dB SPL. **C**: CM evoked by 8 kHz tone bursts with sound intensity varied from 30 to 90 dB SPL. The CM response at 60 dB SPL in B and C is plotted in an expanded scale. Color code: black (WT), blue (heterozygous), red (homozygous) in all subsequent figures.

In the following sets of experiments, we determined whether loss or reduction of an EP have any effect on hair cell survival. We first measured ABR threshold of homozygous and heterozygous *Mitf*-mutant mice and their WT littermates between P30 and P35. Fig 4A shows the mean values of the threshold obtained from six animals for each genotype. No positive ABR response was detected at any frequencies tested at 100 dB SPL in homozygous mice, whereas heterozygous mice displayed some positive ABR responses below 16 kHz at high sound intensity level. Although some hearing remained below 16 kHz, the ABR threshold of heterozygous mice was significantly elevated when compared to that of their WT littermates. We also measured CM evoked by tone bursts at 2 and 8 kHz using the round-window electrode technique. Some examples are shown in Fig 4B and 4C. WT littermates exhibited a CM response with a compound action potential (N1 and N2) at the beginning and end of the CM response. A small action potential (N1) was observed at 90 dB SPL in heterozygous mice. This is consistent with the fact that majority of IHCs were present and functional. Homozygous mice showed no positive response at any sound intensity level used.

**Fig 5:**
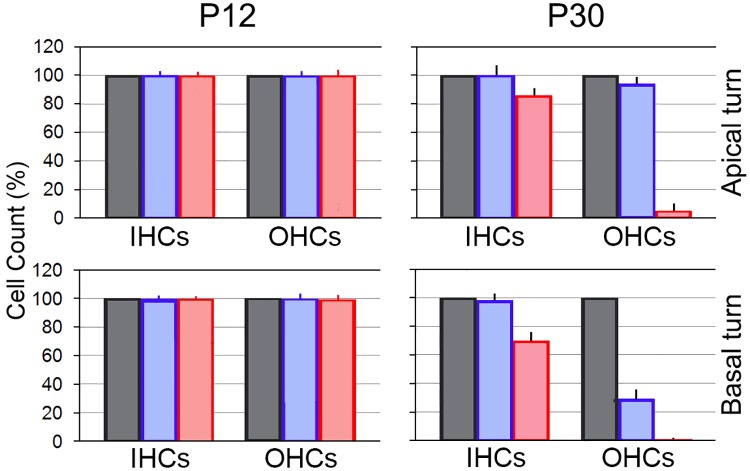
IHC and OHC counts (mean and SD) from four heterozygous and four homozygous mice in two cochlear locations at P12 and P30. IHC and OHC counts obtained from two locations in four WT mice are used as reference (100%).

We used confocal microscopy to examine the status of hair cells in two cochlear locations (5.0–5.4 and 0.2–0.6 mm from the hook region) of *Mitf*-mutant and WT mice at P12 and P30. No obvious IHC or OHC loss in either location in heterozygous or homozygous mice was observed at P12. At P30, there was no obvious IHC loss in either the basal or apical turn location of heterozygous mice. However, substantial OHC loss was observed in the basal turn location. In contrast, OHC loss in the apex was only sporadic. For homozygous mice, OHCs were completely missing in the basal area, though most IHCs remained. In the apical region, most IHCs were present, whereas OHC loss was extensive. We counted the total number of IHCs and OHCs in the two areas from four heterozygous and four homozygous mice. IHC and OHC counts from four WT mice were used as controls (100%). Fig 5 shows the mean and SD from those counts. As shown, hair cell loss is more substantial in homozygous than in heterozygous mice. OHC loss is more dramatic in comparison to IHC loss in both heterozygous and homozygous mice. Hair cell loss in the basal turn is more substantial than hair cell loss in the apical turn.

### Stria morphology and function in *Mitf*-mutant mice

**Fig 6:**
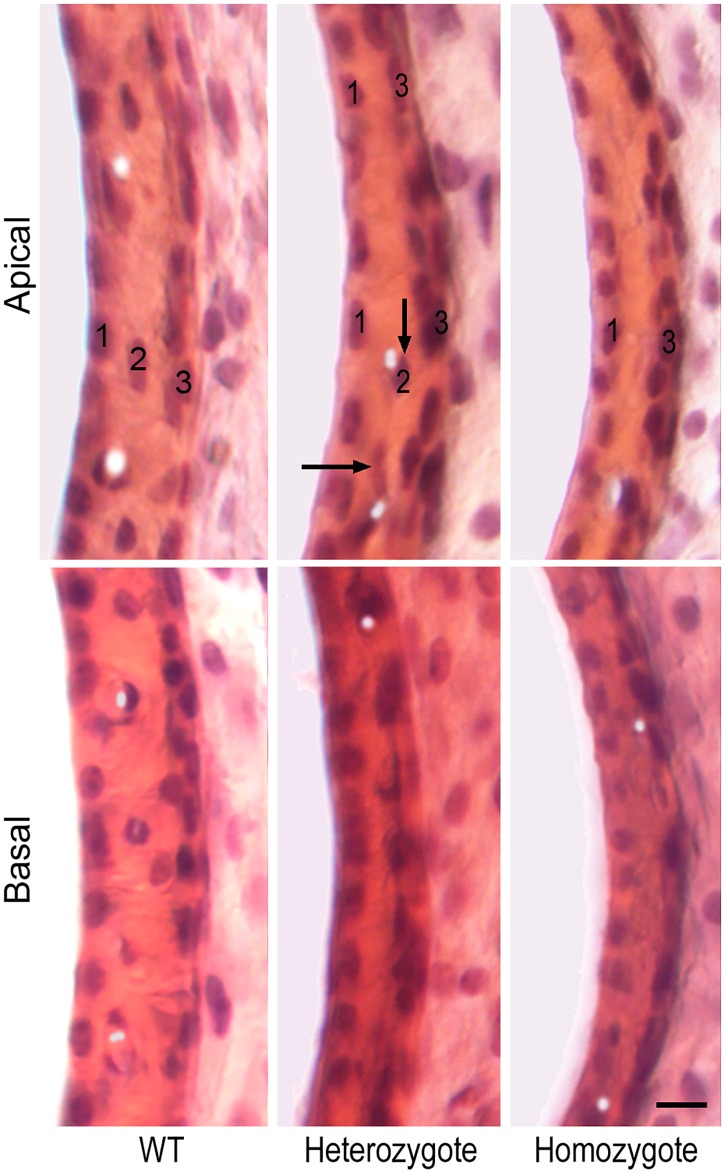
Stria morphology of WT and *Mitf*-mutant mice. Three layers of cells are present in the stria vascularis of WT mice. 1, 2 and 3 mark marginal, intermediate (melanocyte), and basal cells, respectively. Black arrows mark the presence of some melanocytes in the apical turn of a heterozygous mouse. This apical location is a transition region between two and three layers of cells in the stria. Bar represents 20 μm.

We examined stria morphology and function in *Mitf*-mutant mice at P30 using cross sections of the cochlear lateral wall in apical and basal turns. Some examples stained with H&E are exhibited in Fig 6. Three layers of cells (marginal, intermediate or melanocyte, and basal layers) were clearly visible in the stria of both apical and basal turns in WT mice (Fig 6). In the basal turn stria of heterozygous mice, intermediate cells (melanocytes) were no longer present and only two layers of cells were seen. In the apical turn stria of the heterozygous mice, however, some melanocytes were still present (marked by arrows in Fig 6). In homozygous mice, no melanocytes were observed in either apical or basal turn stria (Fig 6).

**Fig 7:**
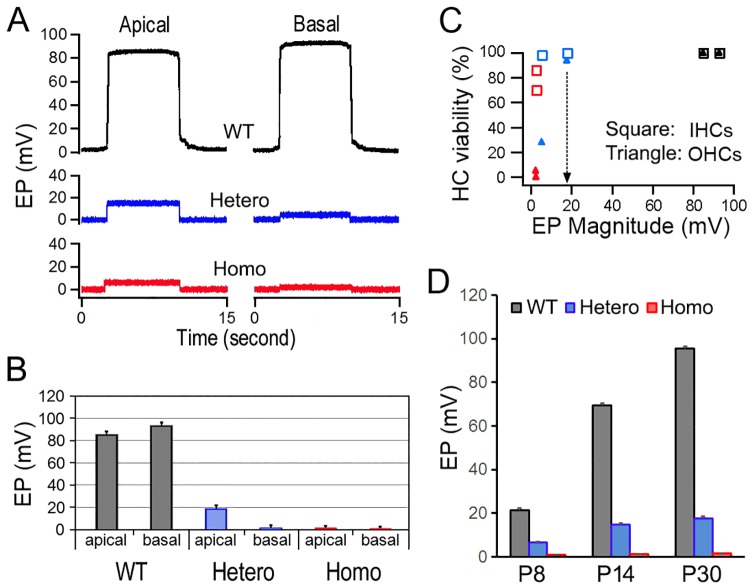
EP measured from apical and basal turns of WT and *Mift*-mutant mice. **A**: Some examples of EP waveforms from WT, heterozygous, and homozygous mice. **B**: Means and SD of EP magnitudes obtained from six mice per mouse strain. **C**: Relationship between EP magnitude and hair cell viability. Hair cell viability (in percentage) is based on cell counts presented in Fig 5B. Open squares represent IHCs, while filled triangles represent OHCs. **D**: EP measured from the basal turn of WT, heterozygous, and homozygous mice at P8, P14, and P30.

We measured the EP in basal and apical turns of *Mitf*-mutant and WT mice. Fig 7A shows some examples of the EPs measured from the two locations in WT, heterozygous, and homozygous mice. Six animals for each genotype were measured. The values of mean and SD are plotted in Fig 7B. The EP magnitude in the apex and base of WT mice was 85.3 ± 3.4 and 94.2 ± 3.5 mV, respectively. In contrast, the EP magnitude was reduced to 18.0 ± 3.3 and 2.3 ± 2.6 mV in the apical and basal turn of heterozygous mice. Although both values were significantly less (*p* = 3.3e-12 and 9.6e-14) than those of WT mice, the reduction in the basal turn was more dramatic. In addition, the EP magnitude in the apical turn was significantly greater (*p* = 1.1e-6) than that in the basal turn; opposite of that seen in WT mice. In homozygous mice, the EP magnitude was reduced to 2.7 ± 2.0 and 1.3 ± 1.4 mV in the apex and base, respectively.

It thus appeared that reduction in the EP magnitude was accompanied by hair cell loss. Fig 7C shows the relationship between the EP magnitude and hair cell viability. It is apparent from the figure that significant OHC loss occurs when the EP magnitude drops below ~18 mV. IHCs were more tolerant of reduction in the EP magnitude, and 60% of IHCs were still viable even when the EP magnitude was reduced to only 2 mV. Thus, survival of OHCs is more vulnerable to reduction of the EP than that of IHCs.

We also examined the development of the EP in *Mitf*-mutant mice at P8, P14, and P30. Fig 7D shows some representative responses from the basal turn of WT, heterozygous, and homozygous mice. The EP magnitude in WT mice increased from ~20 mV at P8 to ~75 mV at P14. By P30, an EP of ~95 mV was observed. In contrast, the EP magnitude was only about 2 mV at P8 and remained at this value between P8 and P30. In other words, the EP in the basal turn of *Mitf*-mutant mice did not develop at all during this time window.

### Examination of OHC function *in vitro*

**Fig 8:**
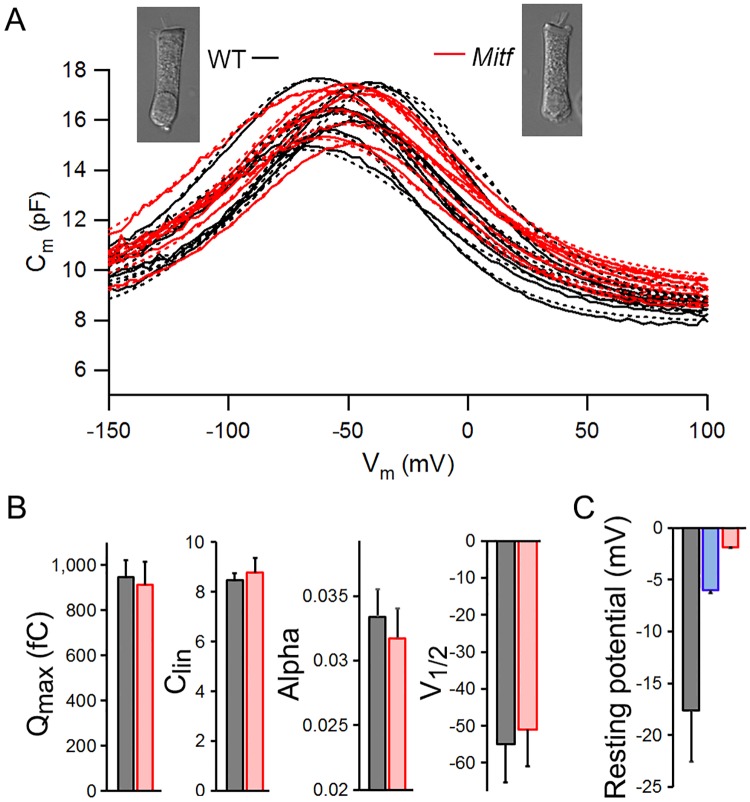
Nonlinear capacitance and membrane potential measured from mice at P21. **A**: Nonlinear capacitance measured from the apical turn OHCs of WT (in black) and *Mift*-mutant homozygous (in red) mice. A first-order Boltzmann function (in dotted lines) was used to compute four parameters that describe the voltage-dependent function of OHC motility. **B**: The computed mean values of four parameters are: *Q*_max_: 946.8 ± 87.4 vs. 911.5 ± 112.1 fC; α: 0.033 ± 0.003 vs. 0.032 ± 0.002; V_1/2_: -54.9 ± 10.9 vs. -51 ± 8.8 mV; *C*_lin_: 8.4 ± 0.34 vs. 8.7 ± 0.88 pF. Comparison of these four parameters between the WT and homozygous OHCs showed no statistical significance (*p* = 0.39, 0.15, 0.25, 0.23, n = 8, respectively). C: Resting membrane potential (mean ± SD) from WT (black), heterozygous (blue) and homozygous (red) mice. *p* = 8.5e-4 between WT and heterozygous OHCs; *p* = 3.1e-5 between WT and homozygous OHCs.

To determine whether OHCs are functional in *Mitf*-mutant mice, we measured their characteristic voltage-dependent motor function [40,41]. Since NLC is regarded as an electrical signature of OHC motility [30,31], we measured NLC from homozygous *Mitf*-mutant OHCs from the apical turn at P21. Fig 8A shows NLC responses obtained from eight WT and eight homozygous OHCs. We computed four parameters from curve fitting of the NLC response by first-order Boltzmann function. These four parameters are generally used to characterize sensitivity and voltage-dependent properties of OHC motility. Comparison of these four parameters (Fig 8B) between WT and homozygous *Mitf*-mutant OHCs showed no significant differences. Thus, the motor function of OHCs in *Mitf*-mutant mice is comparable to that of WT OHCs, when measured under an *in vitro* condition.

Since the electrochemical environment in the endolymph plays an important role in maintaining the resting membrane potential of hair cells, we speculated that the resting membrane potential of hair cells is significantly reduced (depolarized) when the EP and K^+^ concentration in the endolymph are both drastically reduced. Direct measurement of resting membrane potential of hair cells *in vivo* is extremely difficult. Thus, we measured the resting membrane potential of isolated OHCs using the whole-cell voltage-clamp technique as described by other studies [42–44]. Although hair cells are usually depolarized when the electrochemical environment bathing their apical surface is no longer present *in vitro* [42,45], we speculated that the *Mitf*-mutant OHCs would be more depolarized when compared with their WT counterpart. Resting membrane potential in our measurement was defined as the potential observed immediately upon penetrating the cell membrane with the patch electrode [42]. Since the membrane potential would rapidly become more negative as the pipette contents equilibrated with the cell interior over a period of 30–60 seconds, only the initial potential reflects the real resting membrane potential of the cells. Eight OHCs from the apical turn of the each of three genotypes at P21were measured. The mean value of WT, heterozygous, and homozygous OHCs was -17.6 ± 4.9, -6.1 ± 2.2, and -1.9 ± 1.2 mV, respectively (Fig 8C). The resting membrane potential of *Mitf*-mutant OHCs was significantly less than that of the WT control (*p* = 8.5e-4 between WT and heterozygous OHCs, *p* = 3.1e-5 between WT and homozygous OHCs).

## Discussion

The electrochemical environment in the endolymph maintained by the stria has long been known to be critical for hair cell mechanotransduction. However, it is unclear whether the EP is also critical for hair cell survival. Although past studies showed hair cell loss in some animal models that displayed stria defect due to genetic deficiency [16, 21, 46], no studies have explicitly attributed hair cell loss to the loss of the EP. In addition, it is also unclear whether the survival of the stria depends on a functional organ of Corti. In the present study, we determined whether the organ of Corti and stria vascularis depend on each other for survival using genetic mutations that only affect either one or the other organ. Our conclusion represents a new knowledge that is important for understanding the pathology of hearing loss and lays the groundwork for exploring new and practical remedy that may prevent hair cell loss and restore hearing in patients with Waardenburg syndrome. Our novel findings, conclusions, and potential clinical implications are summarized and discussed below:

### Adult OHC survival depends on the electrochemical environment generated and maintained by the stria vascularis and an 18 mV EP may define a threshold for the survival of OHCs

We explicitly showed that adult OHC survival depends on the electrochemical environment generated and maintained by the stria vascularis. This is based on the observation that more than 95% of OHCs were lost when the magnitude of the EP was reduced to near zero millivolt in adult homozygous *Mitf*-mutant mice (Fig 5). IHCs, on the other hand, are less vulnerable to the change in the electrochemical environment in the endolymph. We also observed that a majority of OHCs remained in the apical turn of heterozygous *Mitf*-mutant mice (Fig 5) where an 18-mV EP was still retained (Fig 7). This reduced EP is generated by the remaining intermediate cells in the stria vascularis of the apical turn (Fig 6, marked by arrows). In the basal turn, only a fraction of OHCs remained when the EP was reduced to near zero millivolt. Thus, it appears that while hair cells can tolerate some degree of EP reduction, an 18 mV EP or more is needed for the survival of OHCs. The fact that no hair cell pathology was observed in *Claudin* 11-null mice [47] when the EP was reduced to 30 mV also supports our conclusion that hair cells can tolerate some degree of EP reduction. The EP is never developed in *Mitf*-mutant mice, consistent with a previous study which showed that no intermediate cells were ever present in *Mitf*-mutant homozygous mice [16]. The fact that the EP was never developed in homozygous *Mitf*-mutant mice with no intermediate cells clearly suggests that the EP was generated by the stria.

We observed substantial loss of hair cells (especially OHCs) in *Mitf*-mutant mice. Significant hair cell loss was also observed in *Mitf*-mutant pigs [48]. There are two possibilities that can cause hair cell death in the *Mitf* mutant mice: First, mutation of *Mitf* causes hair cell death if Mitf regulates genes that are important for hair cell function and survival. Second, hair cell loss is caused by loss and reduction of the EP and high K^+^ concentration in the endolymph. Our recent transcriptomic analyses [49,50] show that *Mitf* is expressed in both IHCs and OHCs at very low levels (searchable database available at: https://shield.hms.harvard.edu/gene_search.html). However, it is highly unlikely that hair cell death is caused by loss of Mitf function in hair cells. This is based on the following three reasons: First, the level of *Mitf* expression in adult IHCs and OHCs is about the same (18.14 ± 0.75 for IHCs vs. 15.16 ± 2.26 for OHCs) with no statistical significance [49,50]. If *Mitf* gene were important for hair cell function and survival, loss of IHCs and OHCs would have been similar. The fact that there is a significant difference in loss of hair cells between the two populations suggests that *Mitf* mutation does not directly affect hair cell function/survival. Second, OHC loss was location-dependent, with 76% loss in the basal turn and only 5% loss in the apical turn in heterozygous mice. One would expect to see OHC loss in all turns if the loss were due to loss of function associated with the *Mitf* gene in hair cells. Third and most importantly, OHC loss was minimal when an 18 mV EP was present in the apical turn. All these suggest that OHC death is not caused by loss of function of the *Mitf* gene in hair cells. Instead, OHC death is likely caused by loss of the EP and change (i.e., reduction) in K^+^ concentration in the endolymph. Although we did not measure K^+^ concentration in the endolymph in our study, a recent study has shown that K^+^ concentration in the endolymph is reduced from ~145 mM to ~5 mM in *Mitf*-mutant pigs [48]. The resting membrane potential of hair cells is established by higher concentration of K^+^ (inside the hair cells) and by diffusion of K^+^ across the basolateral membrane. K^+^ influx (leak) through the mechanotransduction channel at rest, together with the Na^+^/K^+^ pump in the basolateral membrane, plays an important role in establishing and maintaining membrane potential of hair cells [42,45]. Reduction of K^+^ influx (due to decrease of EP and/or K^+^ concentration in the endolymph) through transduction channels leads to the reduction of K^+^ concentration gradient across the basolateral membrane, which moves the resting membrane potential toward zero, resulting in Ca^++^ influx, which further depolarizes the cells. Accumulation of Ca^++^ intracellularly can trigger Ca^++^-mediated apoptosis. The resting membrane potential of ~-2 mV seen in *Mitf*-mutant OHCs (Fig 8C) has provided evidence to support this.

### OHC loss in *Mitf*-mutant mice occurs only after P12, a window of opportunity to intervene to prevent OHC loss

Our experiments suggest that the hearing loss in *Mitf*-mutant mice is caused by two mechanisms: loss of the EP and subsequent progressive OHC death from the base to the apex as a result of loss of the EP. Interestingly, OHC loss in the basal turn did not occur before the onset of hearing at P12, suggesting that OHCs in neonatal mice are not so vulnerable to the electrochemical environment in the endolymph (which explains why only neonatal organ of Corti can survive in culture condition for about two weeks). The mechanisms of why neonatal and adult OHCs are subject differently to the EP are unknown. We speculate that functional maturation of mechanotransduction channels and ion channels, especially those involved in maintaining resting membrane potentials (such as Kcnq4 and Kcnj13), may play an important role. It is also unclear why *Mitf* mutation would result in loss (or undifferentiation) of melanocytes. These two questions are important and deserve future investigation. Regardless, this 12-day period before OHCs began to die in neonatal animals offers a window of opportunity to intervene to prevent OHC loss. We speculate that applying a dc potential (i.e., injection of a dc current) in the endolymph to simulate an EP might be a simple and practical strategy to prevent hair cell loss. Since hair cells are still functional (Figs 4C and 8A), this strategy may be able to restore hearing in mice or humans with abnormal stria vascularis (such as in Waardenburg syndrome and Tietz syndrome). The effect of dc current on IHC receptor potential and cochlear harmonics has been studied before [51,52]. It is interesting to note that a small compound action potential (with a small CM) was still observed at low frequency (2 kHz) with high intensity sound in heterozygous *Mitf*-mutant mice (Fig 4C). This suggests that IHCs and OHCs in the apical turn are still functional despite a reduced EP. An ABR threshold elevation between 25 and 50 dB is apparently due to the reduction of the EP (from ~85 mV in WT mice to ~18 mV in heterozygous mice). This is consistent with the level of furosemide-induced reduction of the basilar membrane vibration [12] and threshold elevations in the tuning curves of auditory nerve fibers [13,14]. In homozygous mice, concurrent absence of intermediate cells and the EP further confirms the role of the intermediate cells in generating the EP [2,5,8].

### Function and survival of the stria vascularis do not depend on the presence of a functional organ of Corti

Tasaki and Spyropoulos [1] showed that hair cells in “waltzing guinea pigs” were not needed for the maintenance of the EP, and thus proposed that the EP is generated by the cells in the stria vascularis. Using *Atoh1*-CKO mice, we showed that loss of the organ of Corti has no impact on the survival of the stria vascularis. The morphology of the stria remained normal and its ability to produce an EP was not affected even at 13 months of age, long after all hair cells were gone. The vulnerability of the EP to anoxia and its ability to quickly recover further indicate that the stria vascularis is functional. These observations explicitly demonstrate that the function and survival of the stria vascularis does not depend on the presence of functional hair cells or the supporting cells in the organ of Corti.

It is generally accepted that there is a K^+^ recycling loop that involves hair cells, and a network of supporting cells and fibrocytes, connected by gap junctions along the basilar membrane and lateral wall of the scala media. This K^+^ recycling loop is assumed to be important for maintaining the EP and homeostasis of the ion composition in the endolymph [4]. In the *Atoh1*-CKO mice, this pathway is no longer present since the entire organ of Corti degenerates and the basilar membrane only contains one to two layers of epithelial cells [35]. One would expect that the EP magnitude would drop if this recycling loop is no longer present. However, we showed that the EP remained and the stria vascularis was functional. Therefore, while the present study cannot rule out the existence of this hypothetical loop in the normal cochlea, it is obvious that disruption of this loop (i.e., when hair cells and supporting cells are no longer present) does not affect the production and maintenance of the EP. Strong support for the presence of this K^+^ recycling loop came from studies that used conditional connexin-knockout mice [53,54]. Those studies have provided evidence that connexin-related (Cx26 and Cx30) gap junction channels in supporting cells mediate K^+^ recycling and absorption [3,10]. However, recent transcriptomic analyses of hair cells show that *Gjb2* (encoding Cx26) and *Gjb6* (encoding Cx30), which are components of gap junction channels and crucial cellular structures during development, are also highly expressed in embryonic and adult hair cells [49, 55, 56]. Therefore, it is possible that deletion of *Gjb2* or *Gjb6* may directly lead to abnormal hair cell development and/or death. A recent study has demonstrated the important role of connexin in hair cell development and survival [57]. In light of the latest information, the *Gbj2*- and *Gbj6*-null models are not sufficient to unequivocally support the K^+^ recycle model in the cochlea. We should add that K^+^ cycling is not limited to the loop through the sensory cells and supporting cells. Other studies show that part of the K^+^ current generated by stria vascularis is carried through outer sulcus cells or through Reissner’s membrane [58–64]. It appears that either the Reissner’s membrane route or the Claudius cells after hair cell loss is sufficient to maintain homeostasis in the endolymph and perilymph since there are no outer sulcus cells in *Atoh1*-CKO mice.

Consistent with Davis’s “battery theory” that assumes that two biological batteries (the EP and resting membrane potential of the hair cells), arranged in series, are combined with the mechanotransduction channel in the hair bundle acting as a variable resistor [65], we observed a significant increase in EP magnitude when hair cells and supporting cells of the organ of Corti were all lost in *Atoh1*-CKO mice. A similar increase in the EP magnitude was also reported in C57BL/6 mice during aging [28]. Hair cell loss is considered for this elevation. In the normal cochlea, there is a standing (leak) current through mechanotransduction channels at rest [66,67]. This current reduces the voltage difference between the scala media and scala tympani [68]. Loss of hair cells diminishes this leak current and increases the resistance, which increases the voltage drop (i.e., EP) between the scala media and scala tympani.

In summary, we showed that a complete hair cell loss using *Atoh1*-CKO mice has no short- and long-term impact on the function and survival of the stria. We suggest that the K^+^ recycling route through hair cells and supporting cells is not essential for maintaining the EP. Using a *Mitf*-mutant mouse model in which only the stria was affected, we showed that OHC survival depends on normal function of the stria. We proposed that the hearing loss in *Mitf*-mutant mice is caused by two mechanisms: loss of the EP and subsequent progressive hair cell death from the base to the apex. Such hair cell loss does not occur right after birth. Instead, it occurs after the onset of hearing. This two-week period offers a window of opportunity to intervene. We further showed that OHCs are more vulnerable to the reduction in EP than IHCs. The fact that an 18 mV EP may define a threshold for the survival of OHCs suggests that any intervention by either gene therapy or implanted electrode that can partially rescue stria function is sufficient to prevent loss of hair cells. Our conclusion, based on experiments carried out in two mouse models that affect exclusively either hair cells or stria function, represents a new knowledge that is important for understanding the pathology of hearing loss and lays the groundwork for exploring new and practical remedy that may prevent hair cell loss and restore hearing in patients with Waardenburg syndrome.
